# Cæsarean Operation

**Published:** 1836

**Authors:** Robert Estep

**Affiliations:** Stark county, Ohio


### WESTERN JOURNAL

OF THE

### MEDICAL AND PHYSICAL SCIENCES.

**For April, May and June, 1836.**

### ESSAYS AND CASES.

### Art. I.—Cæsarian Operation.

*The Cæsarian Operation performed twice, successfully, on the same woman.* By Robert Estep, M. D., of Stark county, Ohio.

Aug. 10th, 1830.—I had a hurried call to the wife of Geo. Stull, of Columbiana county. Found the patient of low stature, full habit, a brunette of 20 years of age, in active labor from her first pregnancy. A female attendant informed me, that she had been nearly in that condition for 24 hours. On examination, I found the soft parts well disposed and developed; the position of the head favorable, being the first presentation of the vertex; the membranes ruptured and the waters discharged. Learning that no perceptible progress had been made for the last 24 hours, and finding that mere manual assistance was impracticable, I applied the forceps and delivered the woman of a very small dead fœtus: the head compressed and elongated to a very unusual degree. Suspecting deformity of the pelvis, I took occasion while delivering the placenta, to satisfy myself, and found its anterio-posterior diameter reduced far below any case I had met with; to a degree, indeed, which in my opinion precluded the possibility of her ever giving birth to a living child approaching the ordinary size. This opinion I communicated to the husband, and took my leave. The woman recovered without impediment.

June 22d, 1831. Saw Mrs. S. at the house of a neighbor, and learning that she had by a few days passed the 7th month of pregnancy, advised premature delivery. This expedient was rejected.

Aug. 11th. She was again in labor. The details of the preceding case so perfectly apply to the present, as to deprive it of interest; by extreme throes and the assistance of the forceps, she was delivered of another small, still-born child, with the same distortion of the head. I now made another and more careful examination of the pelvis, and satisfied myself, beyond a doubt, of the abnormal projection of the sacrum, reducing the sacro-pubic diameter below two inches.

Jan. 11th, 1833. Mr. S. called in my absence, and with tears requested my attendance as soon as I returned. On receiving the message I immediately repaired to his house. As soon as I cast my eye upon the patient I observed an inordinate protrusion of the abdomen, which was perceptibly magnified at the accession of every pain. On inquiry I learned of the patient herself, that the membranes broke without any premonitory uneasiness; that during the 3d or 4th pain she distinctly felt "something give way," from which time the swelling of the abdomen became more and more conspicuous. By touching, I found the state of the soft parts and the presentation, as favorable as formerly; but the labor little or nothing advanced, the head resting on the superior straight, and the throes severe, but without any expulsive tendency. From the first half hour I had been in her presence, the unwelcome idea of a rupture of the uterus, had at intervals obtruded itself upon me. At length I gave an opiate, which procured for her some respite, and for me, some time for deliberation. After permitting her to rest for an hour, I commenced a more thorough examination than I had yet made: gently pressing the head of the fœtus back, and resting it on the right iliac fossa, I passed my hand carefully along the anterior portion of the uterus. I had proceded but about mid-way the body of that organ, when I discovered as I thought a longitudinal rent, and its whole anterior aspect distended to a mere membrane. Here was a formidable difficulty—one requiring prompt decision and action. The position of the hand, owing to the extreme contraction of the pelvis, was exceedingly irksome, and viewing the frequent introduction of it as a serious evil, I determined before withdrawing it to turn the fœtus and deliver by the feet, well assured that I could expect no further assistance from the action of the uterus. Pursuing this determination, I grasped both feet, and brought them down: I forbear to subject the patience of the reader to the trials my own, endured in vain endeavors to accomplish the delivery—suffice it to say, that after three hours of indefatigable exertion, I was unable even to get the head engaged in the superior straight. Relinquishing all hope of success by this artifice, and being thoroughly assured of the child's death, I now separated the trunk from the head, in the vague hope of being able to get a better diameter of the latter, or by locking the finger into the chin, to apply a more efficient force; but in this, likewise, I was unsuccessful.

It may be proper here to state, that I was unprovided with any instruments, save a pair of forceps and a common pocket case; nor was there any professional assistant within eight miles, whose counsel I could regard. I had endeavored to perforate the head with a pair of pointed scissors, but the absence of any resisting force defeated the intention. Thinking it possible to succeed with a longer instrument, I went at midnight to a blacksmith's shop, a mile off, and had a rough substitute for a perforator hastily made. It is almost superfluous to add, that the same ill success attended its application.

Seeing my patient rapidly sinking, I now, as a dernier resort, and as the only chance of saving her life, proposed the Cæsarian operation. Contrary to my expectation, she and her friends unhesitatingly consented, and urged its speedy execution. On my part, having for several hours contemplated the matter, I, with as little hesitation, prepared some adhesive strips, lint and bandages, armed a few needles with ligatures, &c., placed the patient in a convenient posture and commenced the operation. My first incision commencing about an inch below and half an inch to the right of the umbilicus, was continued downward, about seven inches, through the integuments only; directing a by-stander to draw the integuments towards the opposite side, by a second incision I divided the linea-alba and peritoneum at the same stroke of the kinfe, guarding the viscera, by elevating the parietes with two fingers of the left hand. The uterus now somewhat contracted, was laid bare, and the fissure perceptible. I brought the organ forward by introducing two fingers within the lacerated wound, which I enlarged with a bistory to the extent of six inches, and grasping the head with the right hand extracted it through the wound. The extremity of the cord still remaining outside the vulva, was now taken hold of, and the placenta delivered by the natural passages. The wound, closed up with sutures and adhesive strips, was dressed with lint, and the patient put to bed, as may be supposed, much exhausted.* Having been exposed to cold, loss of sleep, mental and physical exertion, for the last forty-eight hours, I gave my patient an opiate, and sought repose myself. After a few hours sleep, I was agreeably surprised to find her as much refreshed as myself—cheerful, communicative, and taking nourishment. I gave the necessary directions, and took my leave, with a promise to make a visit early the next day.

13th. Patient worse; considerable fever with full pulse, tender and inflated abdomen. Drew blood copiously, and administered mild enemeta.

---

*Honest criticism may suggest, that, previous to turning, I should have perforated and emptied the cranium. But what assurance had I, that the very next throe might not enlarge the rent, and transfer the contents of the uterus into the abdominal cavity ? My hand was now between the child and the opening; and with such deliberation as the moment allowed me, I deemed it most prudent to secure a foot-presentation, whilst I had the power so to do. Had the head been no larger than that of her preceding children, I yet believe I might have succeeded. Be that as it may, if I have committed an error, I now put it to the only use I can, by faithfully reporting it: and let him who sees profit by it.

14th. Unfavorable symptoms subsided; the lips of the external wound somewhat separated. Shows the value of the two incisions—drew it together with adhesive strips of a better quality.

15th. Patient says she feels able to be up. Lochial discharge natural.

16th. Removed the sutures; patient comfortable.

19th. Wound contracted in length to four inches, and principally healed.

23d. Patient sits up and walks about the room—discontinue my visits.

April 1st, 1834. Saw Mrs. S.: she states that she has again reached the 7th month of pregnancy. The plan of premature delivery is again pressed upon her, but, for reasons that will appear presently, is also again rejected. A neighboring practitioner has insinuated himself into the family, and impressed them with the belief, that he can deliver her without an operation. I am asked whether I can hold out the same encouragement, and reply in the negative. I state decidedly, that no one but myself has had an opportunity of knowing her peculiar conformation; and that except by mutillating the fœtus, or the Cæsarian operation, she can never be delivered of a full-grown child. From the last conversation with Mr. S., together with other facts I had become acquainted with, I was confident the individual to whom I have alluded would be called on at her approaching confinement; and my knowledge of the *man*, as well as the woman with whom he would have to deal, furnished equal certainty, that his visit would be useless, if nothing worse, and that I should be called in at last. So certain was I of all this, that I had every instrument and agent that I thought it possible could be called in requisition, carefully packed up where I could lay my hands on them at any moment. Accordingly, June 2d, I received a hurried call, for which I was prepared. About a mile from town, the husband informed me that Dr. T. was there. On arriving at the house of the patient, I inquired of the attendant what was the prospect? He replied, that there was no alternative but the Cæsarian operation. I demanded of him to state that publicly to the friends, which he forthwith did. I, in the mean time, made examination and found an arm-presentation, and a dead child. The attendant stated it was alive long after he arrived. The patient being clamorous for the operation, and the necessary arrrangements made, was placed in the proper position, and supported by assistants. On examining the old eschar, I discovered that no union of the linea-alba had taken place—I had consequently nothing to do with that tissue. By carefully making an opening for the reception of two fingers of the left hand, and conducting the bistory between them, I completed the incision at a single stroke: on exposing the uterus, its anterior surface was found distended and *transparent* to such a degree, that I could distinguish the members of the fœtus through it, with as much clearness as substances are seen through the coats of an inflated bladder. Continuing the incision through the uterus, I extracted from it a fine, plump full-grown fœtus: the arm, neck and shoulder tumified and discolored, from the position it had occupied. What a pity that ignorant interference should have caused its death. Having divided the cord, I passed its extremity through the wound and os-tincæ, where it was met by a thumb and finger of the other hand passed up the vagina, and again delivered it in the natural way. Clearing the blood from the wound, it was closed with sutures and adhesive strips, dressed with lint and a bandage, and the patient put to bed without any alarming symptom. The loss of blood did not in either of these operations exceed six or eight ounces. It may be observed, that I have made no mention of sutures applied to the wound in the uterus, as recommended by some authors: and I take this occasion to express unqualified disapprobation to their employment. The indissoluble suture, I consider dangerous; the animal ligature, to say the best, useless. By the contraction of the uteres which succeeds parturition, the wound will diminish in a few hours from six or seven, to one or two inches in length; and the viscus itself, from a thin capacious bag, to an almost solid fleshy mass. Indeed, in the last operation, I could discover not a vestige of the former eschar.

June 4th. No discouraging symptoms. 6th. Wound looked healthy—contracted to four inches in length. 8th. Wound healed, patient sits up. 12th. Visited for the last time. She was soon well.

If one example would establish a precedent, this same Cæsarian operation is the easiest mode of bearing children. This woman has ever declared, that the operations were far less severe than the pains of labor.

*March* 20, 1836.

